# Factors Associated with Neural Tube Defects among Women Who Gave Birth at Adama Hospital Medical College, Adama, Ethiopia: A Case Control Study

**DOI:** 10.4314/ejhs.v33i4.9

**Published:** 2023-07

**Authors:** Dereje Tegene, Ephrem Mannekulih

**Affiliations:** 1 Department of Obstetrics and Gynecology, Adama Hospital Medical College, Adama, Ethiopia; 2 Department of Public Health, Adama Hospital Medical College, Adama, Ethiopia

**Keywords:** Adama, Case control study, Neural Tube Defects, Risk Factors

## Abstract

**Background:**

Neural tube defects (NTDs) occur as a result of incomplete closure of the neural tube by the embryonic age of 26 to 28 days. Addressing factors associated with NTDs would help to identify and prioritize high-risk women, which in turn guides the preventive strategy. The study aimed to identify factors associated with NTDs among women who gave birth or had a second-trimester abortion at Adama Hospital Medical College, from January 1st to December 31st, 2019.

**Methods:**

Hospital based unmatched case-control study was conducted on 344 women. Data were analyzed with SPSS 20. Descriptive statistics were computed. Binary logistic regression analysis was performed to determine factors associations with NTDs.

**Results:**

The odds of having a fetus with NTDs were 2.19 times higher among women who are not in a marital relationship (AOR = 2.19; 95% CI: 1.13, 4.25). Women with a previous history of Abortion or stillbirth had 3 fold increased risk of having a fetus with NTDs (AOR = 3.05; 95% CI: 1.58, 5.88). Inadequate housing condition nearly doubles the odds of having a fetus with NTDs (AOR = 1.91; 95% CI: 1.20, 3.04). Folic acid or multivitamin supplementation early in pregnancy reduced the odds of having a fetus with NTDs by 43% (AOR = 0.57; 95% CI: 0.35, 0.91)

**Conclusion:**

Being not in a marital relationship, previous history of abortion or stillbirth, and living in inadequate housing conditions were risk factors for NTDs, while multivitamins or folic acid supplementation was a protective factor.

## Introduction

Neural tube defects (NTDs) are congenital malformations of the brain and spine resulting from incomplete closure of the neural tube by the embryonic age of 26 to 28 days (1, 2). Globally birth defects affect an estimated 1 in 33 infants, account for an estimated 270,000 newborn deaths, and result in 3.2 million birth defect-related disabilities every year (3). The most common serious congenital disorders are congenital heart defects, neural tube defects, and Down syndrome (4). Worldwide, each year approximately300,000 babies are born with NTDs (5), resulting in approximately 88,000 deaths and 8.6 million disability-adjusted life years (DALYs) (6, 7). According to the WHO, almost 94% of severe birth defects occur in low- and middle-resource countries, due often to maternal malnutrition and exposure to teratogenic agents such as alcohol and tobacco (3).

Neural tube defects formally comprise anencephaly, spina bifida, cephalocele, and encephalocele while occult spina bifida is not included among NTD because it is often undetectable or its relation to NTD is uncertain (2, 5). Anencephaly is characterized by the absence of the cranium and telencephalic structures, with the skull base and orbits covered only by angiomatous stroma. Cephalocele is the herniation of meninges through a cranial defect, typically located in the midline occipital region. When brain tissue herniates through the skull defect, the anomaly is termed an encephalocele. Spina bifida is a defect in the vertebrae, typically the dorsal arch, with exposure to the meninges and spinal cord. Most cases are open spina bifida —the defect includes the skin and soft tissues. Herniation of a meningeal sac containing neural elements is termed myelomeningocele. When only a meningeal sac is present, the defect is a meningocele (1, 8).

Because of their significant psychological and economic burdens for patients and their families, NTDs continue to be a major public health issue and are gaining more attention around the world (9). About 50 % of pregnancies complicated by NTD end up in early abortion and one out of four fetuses with NTDs would be stillborn (10). Infants born with NTD die in the first year of their lives and those who survived have permanent disabilities (11, 12). Most of the survivors of NTD need various medical supports, including shunts for hydrocephalus, and orthopedic and urologic treatments (12). The surviving cases would encounter many health problems related to the NTD, this includes; physical disabilities, obesity, bed sores, and heart diseases (13). The etiology of NTDs is multifactorial, and several genetic and environmental risk factors are associated with the development of neural tube defects (14). Socio-demographic factors that increase the risk of NTDs include; Maternal age (15-17), lower maternal educational status (16, 18), and being a rural resident (16). Low socioeconomic status (19-21) and maternal obesity (2, 21), were identified by several studies as important factors associated with increased risk of NTDs.

Maternal obstetric factors associated with increased risk of NTDs mentioned in several studies include; unplanned pregnancy (19, 21, 22), previous history of abortion (20), previous history of stillbirth (15, 19, 20, 23), gender of the fetus (19, 21, 22), previously affected child (23-25), family history of NTDs (17), and multivitamin or folic acid supplementation early in pregnancy (19-23). Smoking and indoor air pollution associated with inadequate housing conditions increase the risk of having a fetus with NTDs (18, 26). In addition, several studies conducted in different parts of the world identified several environmental and genetic factors associated with the occurrence of NTDs, this factor includes; alcohol consumption, cigarette smoking, hyperthermia, medications that disturb folic acid metabolism, diet, and nutritional status, social and economic condition, diseases like diabetes, and folic acid deficiency (2, 18, 27-31).

Despite the high burden of neural tube defects in developing countries including Ethiopia, there was a limitation of information regarding factors associated with NTDs. This study aimed to assess factors associated with Neural tube defects among women who gave birth at AHMC. This information would help healthcare providers and health managers to develop a preventive strategy to reduce the burden of NTDs by focusing on high-risk women.

## Methods and Materials

**Study setting, period, and design**: An unmatched case-control study design was conducted to identify factors associated with NTDs among women who gave birth at Adama Hospital Medical College (AHMC), from January 01 to December 31, 2019. AHMC is the only specialized, referral, and teaching hospital under Oromia regional health bureau and is located in Adama town. Adama is located at 100 K.M, to the southeast of the capital city, Addis Ababa. Currently, the hospital provides health care services to more than 6 million people coming from the catchment area. Obstetrics and gynecology are one of the major departments in the college, providing education to medical students & Obstetrics and Gynecology residents. The department also provides several health care services including; Labor and Delivery, Postnatal care, Antenatal care (ANC), Abortion, Family planning, Gynecologic surgery, and Inpatient care. The total number of deliveries was 800 to 900 deliveries per month.

**Study participants**: All women who gave birth or who had a second-trimester abortion (gestational age greater than 12 weeks), at AHMC were taken as a source population for both cases and controls. The study population includes all women who gave birth at AHMC and all women who had second-trimester Abortions at AHMC from January 1st to December 31st, 2019. After delivery or expulsion, all neonates, stillborn, and second-trimester abortus would be examined by the data collectors for the presence or absence of NTDs. All women whose neonates, stillborn's or second-trimester abortus's had NTDs (i.e anencephaly, spina bifida, cephalocele, or encephalocele) were consecutively included as a case. A woman who gave birth or has second-trimester abortion whose neonates, stillborn's or second-trimester abortus with no neural tube defects, at AHMC, were enrolled in the study as a control. The presence or absence of fetal NTD was confirmed by the data collectors and managing physicians by visual inspection of the fetus after delivery or after second-trimester abortion.

**Exclusion criteria**: All women with first-trimester abortion (gestational age less than or equal to 12 weeks) were excluded from the study since it was difficult to identify the presence of NTD at this gestational age. All neonates or stillborn or second-trimester abortus whose congenital anomaly was difficult to describe as NTDs were also excluded. All mothers who gave birth in another health facility and were referred to AHMC for other reasons were excluded. All women who were critically ill at the time of data collection, and those women who gave birth outside AHMC and referred for other reason was excluded.

**Sample size determination and sampling procedure**: The sample size was calculated using Epi info version 7.2 for unmatched case-control study design, by considering the following statistical assumptions; 80% power of the test, 95% confidence level, 1 to 1 case-to-control ratio, and 10% non-response rate. To determine the required sample size We used place of residence, previous history of stillbirth, unplanned pregnancy, and maternal age older than 35 years were taken as exposure variables. To increase the reliability of the study, we used the largest sample size calculated by considering being a rural resident as a risk factor for NTD based on the previous study done in a similar setting. Accordingly, 42.9 % of women having a fetus with NTDs were from rural areas (exposure among the cases), as compared to 27.9 % of women having a normal fetus from urban areas (exposure among the controls) (19) . A total of 344 women (172 cases and 172 controls) were included in the study. All women whose neonates or stillborn or second-trimester abortus had NTDs were consecutively included as a case until the required sample size was reached. A woman who gave birth or had second-trimester abortion next to the case whose neonate or stillborn or second-trimester abortus had no NTDs was randomly taken as a control.

### Operational Definitions

**Abortus**: after the second trimester of abortion, expelled fetal part whose weight is less than 1000gm is labeled as an abortus.

**Adequate housing condition**: Adequately ventilated house (houses having windows) and having a separate room for the kitchen

**Inadequate housing condition**: having the kitchen in the same living room or absence of adequate ventilation (absence of windows for the living house) during heating or indoor coal combustion

**NTDs**: congenital anomaly of the brain and spine, which includes; anencephaly, spina bifida, cephalocele, or encephalocele

**Data collection tools, procedures, and quality control**: Data was collected by the interviewer administering pretested structured questionnaire and observational checklists. The questionnaire was adapted after reviewing the WHO birth defect surveillance tool and different kinds of literature. The component of the questionnaire was grouped in a logical sequence into parental socio-demographic factors, obstetric and medical factors, and maternal behavioral and social factors. The questionnaire and consent form was translated into the locally spoken languages (Afan Oromo and Amharic languages). The validity of the questionnaires was checked by pretesting on 5% of the sample size at Bishoftu General hospital. Data were collected by four BSc midwives working in the labor ward and Michu clinic. One day of training was given to the data collectors. The data collection process was started after the woman gave birth to a fetus with NTD, as confirmed by the data collector and managing physician. Data were collected by reviewing the women's chart and direct interviews with the mothers. Supervision was carried out by the principal investigator throughout the data collection. All filled questionnaires were checked for completeness, accuracy, and consistency by the principal investigator.

**Data processing and analysis**: Data were coded and entered into Epi info version 7.2, and then exported to SPSS version 20 for analysis. Data cleaning, categorizing, and transforming was performed to make data ready for analysis. The characteristics of cases and controls were described using descriptive statistics like frequencies and cross-tabulations. To identify factors associated with NTDs binary logistic regression model was used. A standard model-building approach was, used to develop a predictive model for NTDs. In the process of building a model, first simple logistic regression analysis was performed to identify those variables that have a crude association with NTD at P-value < 0.25. The selected candidate variables were subjected to a multiple logistic regression model to estimate their adjusted effects on NTDs. The statistical significance of independent variables in the final predictive model of NTD was declared at P-value <0.05. The magnitude of association between independent variables and NTDs was estimated by AOR with 95% CI. Before revealing the estimated associations, the final predictive model was assessed for its goodness of fit using Hosmoer and Lemeshow test. Accordingly, the corresponding p-value for the Hosmer and Lemeshow tests' statistics indicated that the model was well fitted, with a P-value = 0.657. The final fitted model was also assessed for multicollinearity using the Variance Inflation Factor (VIF). Accordingly, the mean VIF value was found to be 1.075, and there was no VIF value above 5 indicating the absence of multi-collinearity among the covariates in the fitted regression model.

**Ethics approval and consent to participate**: Ethical clearance was obtained from the Institutional Review Board of Adama Hospital Medical College. The prepared informed consent was also approved by the Ethical Review Board of the college. Informed written consent was obtained from each study participant after the objectives of the study were explained. The data was also coded and the study participants were not identified by their names.

## Results

**Socio-demographic characteristics of cases and controls**: In the current study, 172 cases and 172 controls were compared across different socio-demographic characteristics. Accordingly, the majority of cases and controls were urban residents. The proportions of women who are not in a marital relationship were higher (22.1%) among cases than among controls (9.3%). Regarding maternal education, 40.3% of women in the cases and 48.3% of women in the control group had secondary education and above. Orthodox Christianity and Muslim were the commonest religion for both cases and controls (Table 1).

**Table 1:** Socio-demographic characteristics of parents who gave birth at Adama Hospitals Medical College from January 1^st^ to December 31^st^ 2019 (n=344)

Characteristics	Cases Number (%)	Controls Number (%)	Total Number (%)
**Maternal Age in Years**			
Less than 20	38(22.1)	42(24.4)	80(23.3)
21-25	64(37.2)	62(36.0)	126(36.6)
26-30	49(28.5)	48(27.9)	97(28.2)
30 and above	21(12.2)	20(11.6)	41(11.9)
**Paternal Age in Years**			
20-29	75(43.6)	78(45.3)	153(44.5)
30-39	78(45.3)	73(42.4)	151(43.9)
40 and above	19(11.0)	21(12.2)	40(11.6)
**Place of Residence**			
Urban	129(75.0)	126(73.3)	255(74.1)
Rural	43(25.0)	46(26.7)	89(25.9)
**Maternal Education**			
No Education	22(12.9)	20(11.6)	42(12.2)
Primary	80(46.8)	69(40.1)	149(43.4)
Secondary	53(31.0)	66(38.4)	119(34.7)
Higher Education	16(9.3)	17(9.9)	33(9.6)
**Paternal Education**			
No Education	16(9.3)	15(8.7)	31(9.0)
Primary	68(39.5)	58(33.7)	126(36.6)
Secondary	65(37.8)	59(34.3)	124(36.0)
Higher Education	23(13.4)	40(23.3)	563(18.3)
**Marital Status**			
In a marital relationship	134(77.9)	158(90.7)	290(84.3)
Not in marital relationship	38(22.1)	16(9.3)	54(15.7)
**Ethnicity**			
Oromo	116(67.4)	104(60.5)	220(64.0)
Amhara	45(262)	49(28.5)	94(27.3)
Gurage	2(1.2)	8(4.7)	10(2.9)
Tigre	4(2.3)	2(1.2)	6(1.7)
Others	5(2.9)	9(5.2)	14(4.1)
**Religion**			
Orthodox	86(50.0)	94(54.7)	180(52.3)
Muslim	66(38.4)	43(25.0)	109(31.7)
Protestant	18(10.5)	33(19.2)	51(14.8)
Catholic	2(1.2)	2(1.2)	4(1.2)

**Obstetrics characteristics of cases and controls**: Regarding the reproductive performance of the participants, 76(44.2%) of the cases and 86(50.0%) of the controls were primigravidas. The previous history of stillbirth or abortion was more prevalent among the cases 37(21.5%) than controls 17(9.9%). Nearly one-third of the cases (33.1%) and half of the controls (49.4%) were supplemented with folic acid or multivitamins early in their pregnancy. In this study, we also noted a higher proportion of NTDs among the male fetus, male to female ratio was 1.4:1. More than half of the women in the cases (54.1%) and 70 (40.7%) of women in the control group reported as their housing condition were inadequate (Table 2).

**Table 2:** Obstetrics characteristics of women who gave birth at Adama Hospital Medical College from January 1^st^ to December 31^st^ 2019 (n=344)

Characteristics	CasesN (%)	ControlsN (%)	TotalN (%)
**Gravidity**			
One	76(44.2)	75(43.6)	162(47.1)
2 to 4	78(45.3)	80(46.5)	153(44.5)
5 and above	18(10.5)	17(9.9)	29(8.4)
**Previous still birth or Abortion**			
Yes	37(21.5)	17(9.9)	54(15.7)
No	135(78.5)	155(90.1)	290(84.3)
**Unplanned pregnancy**			
**Yes**	70(40.7)	44(25.6)	114(33.1)
**No**	102(59.3)	128(74.4)	230(66.9)
**Folic acid or multivitamin supplementation**			
**Yes**	57(33.1)	85(49.4)	142(41.3)
**No**	115(66.9)	87(50.6)	202(58.7)
**Sex of Outcome**			
Male	99(57.6)	84(48.8)	183(0.53)
Female	73(42.4)	88(21.2)	161(0.47)
**Housing condition**			
Adequate	79(45.9)	102(59.3)	181(52.6)
Inadequate	93(54.1)	70(40.7)	163(47.6)

**Factors associated with neural tube defects**: Factors associated with the odds of having NTDs were identified using a binary logistic regression model. In the process of analysis, first simple logistic regression was performed to identify variables that had a crude association with NTDs. At this level, we identified marital status, unplanned pregnancy, previous history of stillbirth or abortion, folic acid or multivitamin supplementation early in pregnancy, housing condition, and sex of the fetus had a crude association with NTDs at P-value < 0.25. The selected candidate variables were subjected to multiple logistic regression analyses to determine their adjusted effect on NTDs after controlling for all possible confounding effects. Accordingly, marital status, previous history of stillbirth or abortion, housing condition, and folic acid or multivitamin supplementation early in pregnancy were significantly associated with NTDs at P-value <0.05 (Table 3).

**Table 3:** Factors associated with NTDs among women who gave birth at Adama Hospitals Medical College from January 1^st^ to December 31^st^ 2019 (n=344)

Characteristics	Cases Number (%)	Controls Number (%)	COR[95%CI]	AOR[95%CI]
**Marital Status**				
In a marital relationship	134(77.9)	158(90.7)	Ref.	Ref.
Not in marital relationship	38(22.1)	16 (9.3)	2.76[1.47, 5.18]*	2.19[1.13, 4.25]**
**Previous Still birth or Abortion**				
Yes	37(21.5)	17(9.9)	2.50[1.35, 4.64]*	3.05[1.58, 5.88]**
No	135(78.5)	155(90.1)	Ref.	Ref.
**Unplanned pregnancy**				
Yes	70(40.7)	44(25.6)	2.00[1.26, 3.16]*	1.56[0.94, 2.59]
No	102(59.7)	128(74.4)	Ref.	Ref.
**Folic acid or multivitamin supplementation**				
**Yes**	57(33.1)	85(49.4)	0.51[0.33, 0.79]*	0.57[0.35, 0.91]**
**No**	115(66.9)	87(50.6)	Ref.	Ref.
**Sex of Outcome**				
Male	99(57.6)	84(48.8)	1.42[0.93, 2.17]*	1.48[0.94, 2.33]
Female	73(42.4)	88(21.2)	Ref.	Ref.
**Housing condition**				
Adequate	79(45.9)	102(59.3)	Ref.	Ref.
Inadequate	93(54.1)	70(40.7)	1.72[1.12, 2.63]*	1.91[1.20, 3.04]**

* P < 0.25

** P < 0.05

The study found that; women who were not in a marital relationship had double-fold increased odds of having a pregnancy complicated by NTDs, compared to their counterparts (AOR = 2.19; 95% CI: 1.13, 4.25). The odds of having a fetus with NTDs were increased by three folds among women with a previous history of Abortion or stillbirth (AOR = 3.05; 95% CI: 1.58, 5.88). As per the finding of the study, being a woman living in inadequate housing condition were associated with 1.91 times higher odds of having a fetus with NTDs compared to women living under adequate housing conditions (AOR = 1.91; 95% CI: 1.20, 3.04). The odds of having a fetus with NTDs were decreased by 43% among women who were supplemented with folic acid or multivitamins early in their pregnancy (AOR = 0.57; 95% CI: 0.35, 0.91) (Table 3).

## Discussion

Neural tube defects are the second most common fetal congenital anomalies and are more prevalent in developing countries, because of low health-seeking behaviors and poor preventive health service coverage. In areas like ours, where there are no widely implemented NTDs preventive strategies, identification of factors that increase the women's risk of having a fetus with NTDs would help healthcare providers to develop and implement preventive strategies that address high-risk women.

As per the finding of this study; women who were not in a marital relationship had 2.2 fold increased risk of having a fetus with NTDs compared to those women who were in a marital relationship. This increased risk of having a pregnancy complicated NTDs among women who were not in a marital relationship can be explained by lack of support from their husbands would make them live under stressful and low socio-economic conditions, which in turn increased the risk of having a fetus with NTDs. Several studies have reported an association between low socio-economic status and the risk for NTDs (19-21), in part due to lack of access or motivation to take folic acid or multivitamins pre-conceptionally and early in pregnancy.

Previous history of Abortion or stillbirth increased the risk of having a fetus with NTDs by three folds. The finding of this study is consistent with the systematic review and meta-analysis conducted in Africa (15). Similarly, an increased risk of NTDs in women with a previous history of stillbirth was reported by a case-control study conducted in Saudi Arabia (23), the Tigray regional state of Ethiopia (19), and north shoa, Ethiopia (20). Numerous investigators have studied prior spontaneous abortions as an etiologic risk factor for NTDs (20, 32). The increased risk of NTDs in woman with previous history of abortion can be explained by the hypothesis developed by Knox and Clarke; it says that residual trophoblastic material (“cell rest”) from an immediately preceding pregnancy may interfere with fetal development in the current pregnancy, resulting in NTDs. It has been hypothesized that trophoblastic material is more likely to be present following a spontaneous abortion than following a full-term pregnancy (32, 33). In addition, poor previous pregnancy outcomes such as miscarriage and stillbirth might be related to inadequate replenishment of nutrients essential for normal fetal development which may last unresolved and impact the current pregnancy.

Inadequate housing condition doubles the risk of having a fetus with NTDs. The finding of this study was consistent with a population-based case-control study conducted in the Shanxi Province of china, which revealed; a 1.6-fold increased risk of NTDs among women who had exposure to indoor coal smoke (18). The other studies in china also found that; the absence of adequate ventilation during heating was significantly associated with an increased risk of having an NTD-affected pregnancy (26). The increased risk of having a fetus with NTDs among women living under inadequate housing conditions can be explained by; toxic pollutants such as carbon monoxide and some toxic heavy metals such as arsenic and lead emitted from domestic coal combustion may be involved in the increased risk of NTDs.

According to the finding of this study, folic acid or multivitamin supplementation early in pregnancy reduced the risk of NTDs by 43 %. Since the trend of pre-conceptional care and folic acid supplementation before pregnancy was very low, the study only evaluated the supplementation started early in pregnancy. The finding of our study was in line with the find of the study done in Saudi Arabia (23), western Iran (25), the systematic review in Africa (15), and the study done in Amhara region Ethiopia (16). Several other pieces of research have convincingly shown that pre-conceptional folic acid supplementation started at least one month before conception and continued to 12 weeks of pregnancy can decrease the risk of first occurrence NTDs by 50% and the risk of recurrence up to 70% if taken at the correct time (34, 35). The exact mechanism by which folate supplementation reduces, or prevents the development of NTDs is not well known. Folate and its synthetic form folic acid is vital for the early development process of a healthy fetus and plays a major role as a co-enzyme in numerous biochemical pathways involving methylation, including the synthesis of DNA, RNA, and certain amino acids.

From this study, we conclude that; being a woman not in a marital relationship, previous history of abortion or stillbirth, and having inadequate housing conditions significantly increased the risk of having a pregnancy complicated by NTDs. On the other hand, folic acid or multivitamin supplementation early in pregnancy reduced the risk of having a fetus with NTDs. So it is very important to supplement folic acid or multivitamins early in pregnancy to reduce the risk of NTDs. In developing countries like Ethiopia where universal supplementation for all pregnant women might be difficult, in a such case focusing on a women having a high risk of developing NTDs are advantageous.
